# Association between serum uric acid and female infertility: a cross-sectional study of National Health and Nutrition Examination Survey (NHANES) 2013–2018

**DOI:** 10.1186/s12905-023-02376-2

**Published:** 2023-05-03

**Authors:** Chen Luo, Haiying Cheng, Xiao He, Xiaojun Tan, Xianghong Huang

**Affiliations:** Center for Reproduction and Genetics, Xiangtan Central Hospital, No.120 Heping Road, Yuhu District, Xiangtan, 411100 Hunan P.R. China

**Keywords:** Female infertility, Serum uric acid, Age, BMI, NHANES

## Abstract

**Background:**

Female infertility is a major problem for women of reproductive-age worldwide. Oxidative stress and inflammation are involved in processes related to female infertility. Serum uric acid levels, an indicator of oxidative stress and inflammation, have rarely been reported to be associated with female infertility. This study aimed to investigate the relationship between serum uric acid levels and female infertility.

**Methods:**

This cross-sectional study included women aged 18–44 years from the National Health and Nutrition Examination Survey (NHANES) between 2013 and 2018. All data were extracted from NHANES questionnaires and laboratory measurements. Weighted univariable and multivariable logistic regression analyses were utilized to explore the relationship between serum uric acid and female infertility. Stratified analyses were performed based on body mass index (BMI, < 25 kg/m^2^ and ≥ 25 kg/m^2^) and age (≤ 30 years and > 30 years). The odds ratio (OR) with 95% confidence interval (CI) was used to report associations.

**Results:**

A total of 2,884 women were included, of which 352 (13.30%) had infertility. Women with high serum uric acid concentrations were related to higher odds of infertility (OR = 1.20, 95%CI: 1.03–1.39) after adjusting for confounders. Compared with serum uric acid concentrations ≤ 3.72 mg/dL, women with uric acid concentrations of 4.43–5.13 mg/dL (OR = 1.65, 95%CI: 1.02–2.67) and > 5.13 mg/dL (OR = 1.86, 95%CI: 1.10–3.13) were related to higher odds of infertility. Stratified analyses showed that high serum uric acid concentrations were associated with higher odds of infertility in women with a BMI < 25 kg/m^2^ (OR = 1.41, 95%CI: 1.04–1.93), but not in women with a BMI ≥ 25 kg/m^2^ (*P* = 0.056). In addition, high serum uric acid concentrations were associated with higher odds of infertility in women aged > 30 years (OR = 1.23, 95%CI: 1.04–1.45), but not in women aged ≤ 30 years (*P* = 0.556).

**Conclusion:**

Women with high serum uric acid concentrations were associated with higher odds of infertility, and this association may vary by BMI and age.

## Background

Infertility is a disease characterized by an inability to determine clinical pregnancy after at least 12 months of regular, unprotected sexual intercourse [1]. Infertility has become a public health problem worldwide, affecting an estimated 8–12% of reproductive-aged couples [2]. A global burden analysis of infertility showed that from 1990 to 2017, the age-standardized prevalence of infertility increased by 0.370% per year among women and 0.291% per year among men [3]. Infertility can cause severe psychological and social distress as well as a considerable financial burden to the patient [4, 5]. Female infertility not only affects the reproductive needs of patients, but may also increase the risk of reproductive cancers and metabolism-related diseases [6, 7].

The most common physiological causes of female infertility include ovulatory dysfunction and tubal diseases such as polycystic ovary syndrome (PCOS) and endometriosis [8–10]. Several studies have reported that oxidative stress and inflammation play a significant role in female infertility [11–13]. Oxidative stress may lead to oocyte aging and reproductive pathologies, such as abnormal follicular atresia, abnormal meiosis, reduced fertilization rate, delayed embryonic development, and reproductive system diseases [11]. Inflammation may be involved in the ovulation process and plays a key role in ovarian follicle dynamics [12]. Uric acid is the end product of purine metabolism, and serum uric acid level has been reported to be an important marker of oxidative stress and inflammation in the body [14, 15]. Previous studies have shown that uric acid may be involved in various pathological processes dominated by oxidative stress, and as a pro-inflammatory factor led to systemic inflammation, thereby affecting the related processes of female infertility [9, 16]. In addition, epidemiological evidence suggested that serum uric acid levels were also higher in patients with PCOS [17, 18]. However, the relationship between serum uric acid levels and female infertility has rarely been reported.

Thus, this study aimed to explore the relationship between serum uric acid levels and female infertility. Serum uric acid level may be an indicator of female infertility.

## Methods

### Data source and participants

Data for this cross-sectional study were obtained from the 2013–2018 cycles of the National Health and Nutrition Examination Survey (NHANES). NHANES is a national survey conducted every two years by the National Center for Health Statistics (NCHS) to collect data on the health and nutritional status of the civilian, non-institutionalized population of the United States [19]. NHANES uses a multistage complex stratified sampling method to collect representative samples, and the data is collected through a combination of questionnaires, physical examinations, and laboratory tests. Participants were included if they met the following criteria: (1) women aged 18–44 years; (2) women with measured serum uric acid; (3) women with infertility status information. The exclusion criteria were as follows: (1) pregnant women; (2) women with no sexual experience; (3) women with both ovaries removed; (4) women with a history of hysterectomy; (5) women with missing important covariates. The requirement of ethical approval for this study was waived by the Institutional Review Board of Xiangtan Central Hospital, because the data was accessed from NHANES (a publicly available database). Written informed consent was not required as this study was based on publicly available data. All methods were performed in accordance with the relevant guidelines and regulations.

### Measurement of infertility

The outcome variable of this study was infertility. Infertility status was measured by each woman’s self-reported based on the two questions of the NHANES Reproductive Health Questionnaire (RHQ): (1) item RHQ074, “Have you ever attempted to become pregnant over a period of at least a year without becoming pregnant”; (2) item RHQ076, “Have you ever been to a doctor or other medical provider because you have been unable to become pregnant”. Women who answered “yes” to either of these two questions were considered to have a history of infertility [20].

### Measurement of serum uric acid

The timed endpoint method was used to measure serum uric acid concentration by Beckman Coulter UniCel® DxC800 Synchron Clinical System. The hydrogen peroxide reacts with 4-aminoantipyrine (4-AAP) and 3, 5-dichloro-2-hydroxybenzene sulfonate (DCHBS) under the catalysis of peroxidase to form colored products. The system monitors the change in absorbance at 520 nm at regular time intervals, which is proportional to the concentration of uric acid in the sample. The detailed uric acid testing procedure is presented in the NHANES Laboratory Procedures Manual [21].

### Covariates

Participants’ demographics, medical history, and medication use were collected, including age, race/ethnicity (Mexican American, other Hispanic, non-Hispanic white, non-Hispanic black, other race), poverty to income ratio (≤ 1.0, 1.0–2.0, > 2.0, unknown), education level (less than high school, high school, more than school), marital status (married, never married, others, unknown), smoking (no, ever smoking, smoking regularly), drink alcohol (no, sometimes, regularly), physical activity (< 450 met*min/week, ≥ 450 met*min/week, unknown), age at menarche, menstrual cycle regularity (no, yes), pelvic infection (no, yes), gout (no, yes), hypertension (no, yes), diabetes mellitus (no, yes), dyslipidemia (no, yes), cardiovascular disease (no, yes), body mass index (BMI, < 25 kg/m^2^, ≥ 25 kg/m^2^), hypersensitive C-reactive protein (< 7.48 mg/L, ≥ 7.48 mg/L, unknown), oral contraceptives (no, yes), hormones drug (no, yes), steroid drugs (no, yes), and urate-lowering drugs (no, yes). All these variables were extracted from the records of the NHANES questionnaires and laboratory measurements.

### Statistical analysis

Kolmogorov-Smirnov was used to test the normality of the quantitative data. Normally distributed quantitative data were described as a mean and standard error [mean (S.E)], and the independent samples t-test was used for comparison between two groups, and the analysis of variance (ANOVA) was used for comparison between multiple groups. Non-normal quantitative data were described as median and quartiles [M (Q1, Q3)], and the Kruskal-Wallis test was used for comparisons between multiple groups. Count data were described as numbers and percentages [n (%)], and the Chi-square test or rank sum test was used for comparison between groups. Variables with more missing values (≥ 7%), such as poverty to income ratio, marital status, C-reactive protein, and physical activity, were categorized as “unknown”, while variables with fewer missing values (< 7%), such as smoking, drinking, and pelvic infection, were filled using the random forest multiple imputation method. Sensitivity analysis was performed to compare the difference before and after data imputation. The uric acid concentrations were analyzed in continuous and quartiles (≤ 3.72 mg/dL, 3.72-4.43 mg/dL, 4.43-5.13 mg/dL, and > 5.13 mg/dL), respectively. Weighted univariable logistic regression analysis was used to screen covariates, and variables with statistically significant were included in multivariable logistic regression analysis. Three models were established to evaluate the association between uric acid and infertility. Model 1 was a univariable logistic regression model, model 2 was a multivariable logistic regression model adjusted for age and race/ethnicity, and model 3 was a multivariable logistic regression model adjusted for age, race/ethnicity, marital status, pelvic infection, gout, hypertension, diabetes mellitus, dyslipidemia, BMI, hypersensitive C-reactive protein, and oral contraceptives. The odds ratio (OR) with 95% confidence interval (CI) was used to report associations. Stratified analyses were performed based on BMI (< 25 kg/m^2^ and ≥ 25 kg/m^2^) and age (≤ 30 years and > 30 years) [22].

All analyses were performed by SAS software, version 9.4 (SAS Institute Inc., Cary, NC, USA). A *P* value (two-sided) of < 0.05 was considered statistically significant.

## Results

### Characteristics of participants

**Fig. 1:**
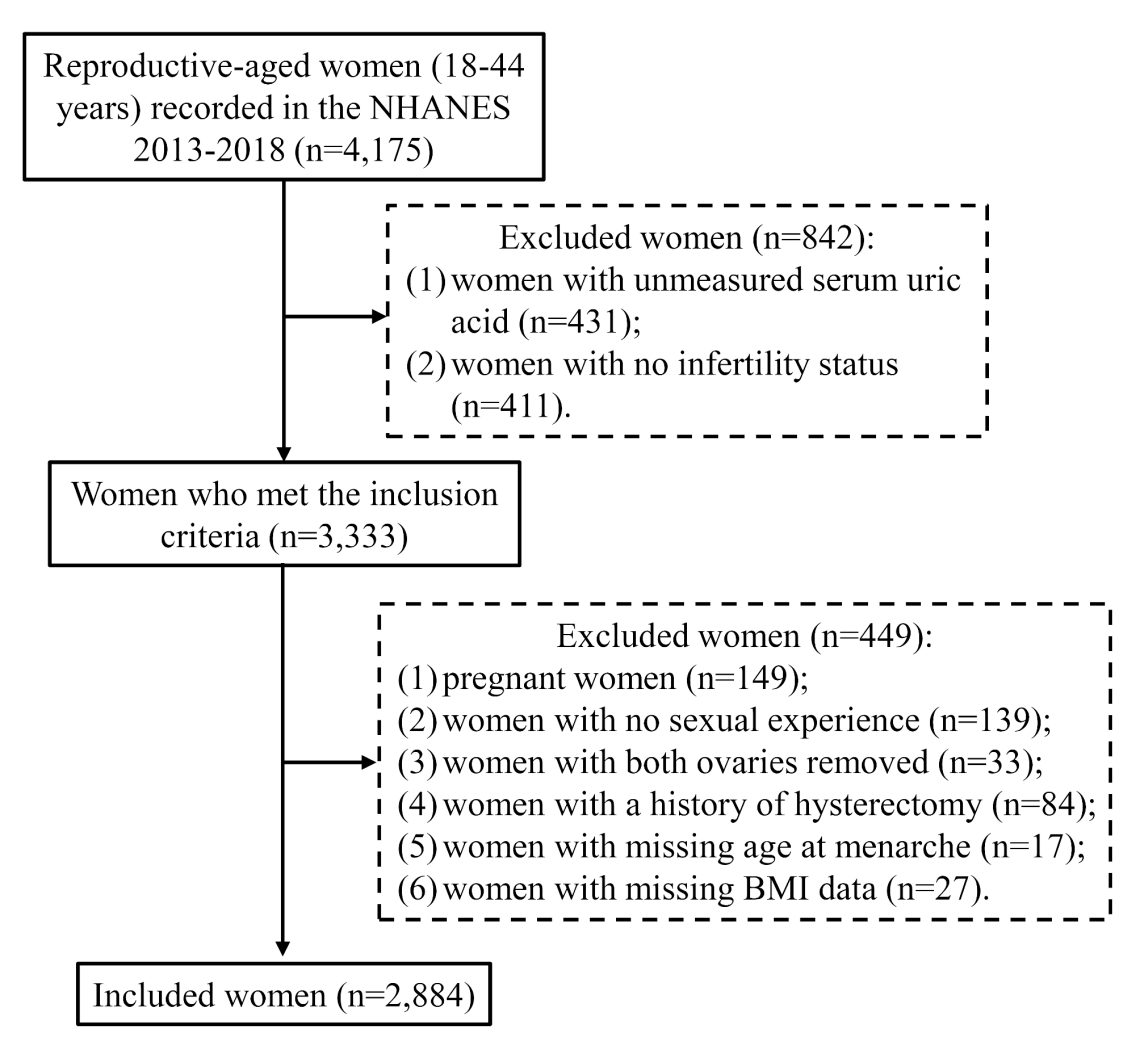
The flow chart of participants inclusion. NHANES, National Health and Nutrition Examination Survey; BMI, body mass index

A total of 4,175 women aged 18–44 years were recorded in the NHANES database from 2013 to 2018. There were 1,291 women excluded, including 431 women with unmeasured serum uric acid, 411 women with no infertility status, 149 pregnant women, 139 women with no sexual experience, 33 women with both ovaries removed, 84 women with a history of hysterectomy, 17 women with missing age at menarche, and 27 women with missing BMI data. A total of 2,884 women were included in this study (Fig. 1). Of the women included, 352 (13.30%) women had infertility (Table 1). The mean (S.E) serum uric acid concentration was 4.56 (0.03) mg/dL. The mean (S.E) age of these women was 30.96 (0.24) years, and 1,819 (61.95%) women had a BMI ≥ 25 kg/m^2^. There were 499 (19.19%) women who smoked, 677 (29.77%) women who drank alcohol regularly, 2,695 (92.78%) women who had a regular menstrual cycle, 134 (4.37%) women who had a pelvic infection, and 1,903 (73.30%) women who took oral contraceptives.

**Table 1:** Characteristics of included women

Variables	Total (n = 2884)	Non-infertility (n = 2532)	Infertility (n = 352)	*P*
Serum urine acid, mg/dL, Mean (S.E)	4.56 (0.03)	4.52 (0.03)	4.79 (0.08)	0.004
Serum urine acid (quartiles), n (%)				0.022
≤3.72 mg/dL	706 (24.38)	643 (25.46)	63 (17.38)	
3.72-4.43 mg/dL	741 (24.63)	658 (24.90)	83 (22.84)	
4.43-5.13 mg/dL	692 (25.15)	608 (24.92)	84 (26.65)	
>5.13 mg/dL	745 (25.84)	623 (24.72)	122 (33.14)	
Age, years, Mean (S.E)	30.96 (0.24)	30.43 (0.24)	34.45 (0.57)	< 0.001
Race/ethnicity, n (%)				0.266
Mexican American	514 (12.31)	454 (12.49)	60 (11.11)	
Other Hispanic	311 (8.24)	286 (8.60)	25 (5.84)	
Non-Hispanic White	942 (55.87)	814 (55.14)	128 (60.65)	
Non-Hispanic Black	616 (12.81)	538 (12.83)	78 (12.72)	
Other races	501 (10.77)	440 (10.94)	61 (9.67)	
Poverty-to-income ratio, n (%)				0.029
≤1.0	711 (19.31)	636 (19.77)	75 (16.33)	
1.0–2.0	701 (21.03)	619 (21.30)	82 (19.30)	
>2.0	1245 (52.86)	1069 (51.73)	176 (60.28)	
Unknown	227 (6.79)	208 (7.20)	19 (4.10)	
Education level, n (%)				0.708
Less than high school	475 (11.86)	420 (12.04)	55 (10.65)	
High school	629 (21.35)	562 (21.50)	67 (20.37)	
More than high school	1780 (66.79)	1550 (66.46)	230 (68.98)	
Marital status, n (%)				< 0.001
Married	1125 (42.33)	920 (39.14)	205 (63.13)	
Never married	818 (28.94)	755 (31.09)	63 (14.97)	
Others	654 (22.81)	575 (23.04)	79 (21.32)	
Unknown	287 (5.91)	282 (6.73)	5 (0.59)	
Smoking, n (%)				0.221
No	2094 (68.93)	1863 (69.68)	231 (64.08)	
Ever smoking	291 (11.88)	244 (11.46)	47 (14.58)	
Smoking regularly	499 (19.19)	425 (18.86)	74 (21.35)	
Drink alcohol, n (%)				0.829
No	790 (21.46)	703 (21.68)	87 (19.99)	
Sometimes (< once a week)	1417 (48.77)	1239 (48.75)	178 (48.90)	
Regularly (≥ once a week)	677 (29.77)	590 (29.56)	87 (31.11)	
Physical activity, n (%)				0.346
<450 met*minutes/week	246 (7.34)	221 (7.38)	25 (7.11)	
≥450 met*minutes/week	2023 (74.32)	1785 (74.79)	238 (71.20)	
Unknown	615 (18.34)	526 (17.83)	89 (21.69)	
Age at menarche, years, Mean (S.E)	12.59 (0.04)	12.60 (0.04)	12.50 (0.13)	0.443
Menstrual cycle regularity, n (%)				0.245
No	189 (7.22)	168 (6.93)	21 (9.10)	
Yes	2695 (92.78)	2364 (93.07)	331 (90.90)	
Pelvic infection, n (%)				< 0.001
No	2750 (95.63)	2435 (96.53)	315 (89.72)	
Yes	134 (4.37)	97 (3.47)	37 (10.28)	
Gout, n (%)				0.021
No	2872 (99.61)	2524 (99.74)	348 (98.81)	
Yes	12 (0.39)	8 (0.26)	4 (1.19)	
Hypertension, n (%)				< 0.001
No	2418 (85.14)	2154 (86.49)	264 (76.31)	
Yes	466 (14.86)	378 (13.51)	88 (23.69)	
Diabetes, n (%)				< 0.001
No	2691 (94.33)	2379 (94.96)	312 (90.23)	
Yes	193 (5.67)	153 (5.04)	40 (9.77)	
Dyslipidemia, n (%)				< 0.001
No	1608 (55.57)	1449 (57.44)	159 (43.39)	
Yes	1276 (44.43)	1083 (42.56)	193 (56.61)	
Cardiovascular disease, n (%)				0.811
No	2768 (95.92)	2431 (95.96)	337 (95.64)	
Yes	116 (4.08)	101 (4.04)	15 (4.36)	
BMI, n (%)				0.005
<25 kg/m^2^	1065 (38.05)	966 (39.40)	99 (29.27)	
≥25 kg/m^2^	1819 (61.95)	1566 (60.60)	253 (70.73)	
Hypersensitive C-reactive protein, n (%)				0.028
<7.48 mg/L	1539 (55.04)	1375 (56.26)	164 (47.05)	
≥7.48 mg/L	313 (11.50)	269 (11.01)	44 (14.75)	
Unknown	1032 (33.46)	888 (32.73)	144 (38.20)	
Oral contraceptives, n (%)				0.041
No	981 (26.70)	889 (27.54)	92 (21.20)	
Yes	1903 (73.30)	1643 (72.46)	260 (78.80)	
Hormones drug, n (%)				0.142
No	2485 (82.76)	2165 (82.07)	320 (87.25)	
Yes	399 (17.24)	367 (17.93)	32 (12.75)	
Steroid drugs, n (%)				0.196
No	2850 (98.64)	2502 (98.54)	348 (99.27)	
Yes	34 (1.36)	30 (1.46)	4 (0.73)	
Urate-lowering drugs, n (%)				0.175
No	2881 (99.94)	2530 (99.96)	351 (99.82)	
Yes	3 (0.06)	2 (0.04)	1 (0.18)	

Note: BMI, body mass index; Mean (S.E); mean and standard error; n (%), numbers and percentages

Compared with non-infertility women, women with infertility had higher serum uric acid concentration (*P* = 0.004), older age (*P* < 0.001), a higher proportion of pelvic infection (*P* < 0.001), gout (*P* = 0.021), hypertension (*P* < 0.001), diabetes mellitus (*P* < 0.001), dyslipidemia (*P* < 0.001), BMI ≥ 25 kg/m^2^ (*P* = 0.005), and oral contraceptives use (*P* = 0.041). Moreover, there were differences between infertility and non-infertility women in the distribution of poverty to income ratio (*P* = 0.029), marital status (*P* < 0.001), and C-reactive protein (*P* = 0.028) (Table 1).

## Association between serum uric acid and infertility

Table 2 reports the association between serum uric acid concentrations and infertility. Women with high serum uric acid concentrations were associated with higher odds of infertility (OR = 1.24, 95%CI: 1.08–1.42). After adjusting for covaries such as age, race/ethnicity, marital status, pelvic infection, gout, hypertension, diabetes mellitus, dyslipidemia, BMI, hypersensitive C-reactive protein, and oral contraceptives, women with high uric acid concentrations were related to higher odds of infertility (OR = 1.20, 95%CI: 1.03–1.39). Compared with serum uric acid concentrations ≤ 3.72 mg/dL, women with uric acid concentrations of 4.43–5.13 mg/dL (OR = 1.65, 95%CI: 1.02–2.67) and > 5.13 mg/dL (OR = 1.86, 95%CI: 1.10–3.13) were associated with higher odds of infertility after adjusting for above covaries.

**Table 2:** Association between serum uric acid concentrations and female infertility

Variables	Model 1		Model 2		Model 3	
	OR (95%CI)	*P*	OR (95%CI)	*P*	OR (95%CI)	*P*
Serum urine acid, mg/dL	1.24 (1.08–1.42)	0.003	1.26 (1.09–1.45)	0.002	1.20 (1.03–1.39)	0.019
Serum urine acid (quartiles)						
≤ 3.72 mg/dL	Ref		Ref		Ref	
3.72-4.43 mg/dL	1.34 (0.81–2.24)	0.252	1.43 (0.86–2.39)	0.167	1.41 (0.85–2.34)	0.182
4.43-5.13 mg/dL	1.57 (1.00-2.46)	0.052	1.73 (1.08–2.78)	0.024	1.65 (1.02–2.67)	0.041
> 5.13 mg/dL	1.96 (1.23–3.15)	0.006	2.09 (1.27–3.44)	0.005	1.86 (1.10–3.13)	0.021

Note: OR, odds ratio; 95%CI, 95% confidence interval; Ref, reference

Model 1: univariable logistic regression model;

Model 2: multivariable logistic regression model adjusted for age and race/ethnicity;

Model 3: multivariable logistic regression model adjusted for age, race/ethnicity, marital status, pelvic infection, gout, hypertension, diabetes mellitus, dyslipidemia, BMI, hypersensitive C-reactive protein, and oral contraceptives

Table 3 shows a stratified analysis of the association between serum uric acid and infertility based on BMI and age. After adjusting for confounders, high serum uric acid concentrations were associated with higher odds of infertility in women with a BMI < 25 kg/m^2^ (OR = 1.41, 95%CI: 1.04–1.93), but not in women with a BMI ≥ 25 kg/m^2^ (*P* = 0.056). Among women with different uric acid concentrations, only uric acid concentrations of 4.43-5.13 mg/dL in women with BMI < 25 kg/m^2^ (OR = 3.06, 95%CI: 1.26–7.43) and > 5.13 mg/dL in women with BMI ≥ 25 kg/m^2^ (OR = 1.78, 95%CI: 1.04–3.05) were related to higher odds of infertility. For women of different ages, high serum uric acid concentrations were related to higher odds of infertility in women aged > 30 years (OR = 1.23, 95%CI: 1.04–1.45), but not in women aged ≤ 30 years (*P* = 0.556). In the analysis of different uric acid concentrations, only uric acid concentrations of 4.43-5.13 mg/dL (OR = 2.14, 95%CI: 1.18–3.89) and > 5.13 mg/dL (OR = 1.94, 95%CI: 1.10–3.39) in women aged > 30 years were associated with higher odds of infertility.

**Table 3:** Stratified analysis of the association between serum uric acid and female infertility based on BMI and age

Variables	Number	OR (95%CI)	*P*	Number	OR (95%CI)	*P*
**BMI subgroup**		BMI < 25 kg/m^2^ (n = 1065)			BMI ≥ 25 kg/m^2^ (n = 1819)	
Serum urine acid (mg/dL)		1.41 (1.04–1.93)	0.030		1.17 (1.00-1.38)	0.056
Serum urine acid (quartiles)						
≤ 3.72 mg/dL	373	Ref		333	Ref	
3.72-4.43 mg/dL	332	1.86 (0.82–4.21)	0.133	409	1.23 (0.66–2.28)	0.503
4.43-5.13 mg/dL	231	3.06 (1.26–7.43)	0.015	461	1.32 (0.78–2.23)	0.293
> 5.13 mg/dL	129	1.55 (0.56–4.34)	0.393	616	1.78 (1.04–3.05)	0.035
**Age subgroup**		Age ≤ 30 years (n = 1398)			Age > 30 years (n = 1486)	
Serum urine acid (mg/dL)		1.11 (0.78–1.59)	0.556		1.23 (1.04–1.45)	0.016
Serum urine acid (quartiles)						
≤ 3.72 mg/dL	327	Ref		379	Ref	
3.72-4.43 mg/dL	354	2.07 (0.66–6.44)	0.205	387	1.22 (0.65–2.27)	0.529
4.43-5.13 mg/dL	355	0.95 (0.25–3.61)	0.940	337	2.14 (1.18–3.89)	0.014
> 5.13 mg/dL	362	1.73 (0.46–6.47)	0.405	383	1.94 (1.10–3.39)	0.022

Note: OR, odds ratio; 95%CI, 95% confidence interval; Ref, reference; BMI, body mass index. All OR were results of a multivariable logistic regression model adjusted for age (not in age subgroup), race/ethnicity, marital status, pelvic infection, gout, hypertension, diabetes mellitus, dyslipidemia, BMI (not in BMI subgroup), hypersensitive C-reactive protein, and oral contraceptives

## Discussion

Infertility is an important challenge for many adult women. This study investigated the association between serum uric acid concentrations and female infertility. The results found that women with high serum uric acid concentrations were associated with higher odds of infertility. Stratified analysis showed that the association between uric acid and infertility in women may vary by BMI and age. Only uric acid concentrations of 4.43-5.13 mg/dL in women with BMI < 25 kg/m^2^ and > 5.13 mg/dL in women with BMI ≥ 25 kg/m^2^ were associated with higher odds of infertility. And the association between uric acid and infertility was found only in women aged > 30 years.

A systematic review reported that the most common causes of infertility are ovulation dysfunction, male factor infertility, and tubal disease [8]. Lifestyle and environmental factors, such as smoking and obesity, can adversely affect fertility [8]. The current study explored the effect of serum uric acid on female infertility. The results found that women with high serum uric acid concentrations were associated with higher odds of infertility. Several studies have summarized the link between uric acid and female reproductive disorders [9, 16]. The possible mechanisms by which serum uric acid levels affect female infertility were as follows. The most common cause of anovulation is PCOS, which affects 80% of women with anovulation [10]. The main pathological changes of PCOS are hyperandrogenemia and insulin resistance [23, 24]. Chronically high uric acid levels can promote hyperandrogenemia, insulin resistance, abnormal lipid metabolism, and complications of PCOS, with potential mechanisms involving oxidative stress, chronic inflammation, and mitochondrial dysfunction [9, 16]. High serum uric acid levels in patients with PCOS are associated with androgen excess and insulin resistance. Androgens may increase serum uric acid levels by inducing hepatic metabolism of purine nucleotides and enhancing purine renewal in the kidney [25, 26]. Under the action of insulin, nitric oxide (NO) is required for glucose uptake and utilization, and uric acid affects the mechanisms associated with insulin resistance by blocking the biological effects of NO and interfering with endothelial function [27]. Excessive production of androgens by the ovaries and adrenal glands can cause massive follicular atresia, ultimately resulting in ovulation disorders in women with PCOS [28]. Moreover, uric acid may be involved in the development of endometriosis through interleukin-1β (IL-1β) [9, 29]. Uric acid is a pro-inflammatory factor that triggers IL-1β-mediated inflammation through activation of the NOD-like receptor protein (NLRP) inflammasome, which is central to many pathological inflammatory conditions [9, 30]. Ma et al. reported that high serum uric acid levels may also affect the decrease in semen quality [31]. High uric acid levels may affect sperm function by altering reproductive hormone levels, altering oxidative stress levels, and disrupting endothelin/nitric oxide balance (leading to endothelial dysfunction) [31].

Studies on the relationship between uric acid and female infertility are sparse. Only one recent study by Liang et al. has reported the association between serum uric acid and female infertility [32]. Consistent with our results, Liang et al. also supported that high serum uric acid levels were related to higher odds of female infertility. Our study differs from that of Liang et al. in the study population criteria and confounders. This further established the association between serum uric acid and female infertility. Previous studies found that age and obesity are also important factors in female infertility [2, 33]. Therefore, we further analyzed the association between uric acid and female infertility based on age and BMI. Our study found that high serum uric acid concentrations were related to higher odds of infertility in women with BMI < 25 kg/m^2^, whereas only the fourth quartile of serum uric acid concentration was related to infertility in women with BMI ≥ 25 kg/m^2^. This result may be related to the fact that obese women have higher serum uric acid levels than normal women [34, 35], which may reduce the sensitivity to the effects of serum uric acid. Broughton et al. reported that obese women are more likely to have ovulation dysfunction due to dysregulation of the hypothalamic-pituitary-ovarian axis [33]. In addition, a prospective cohort study showed that girls who were obese at ages 7–11 were more likely to report infertility in adulthood compared to girls of normal weight [36]. Age has a significant impact on a woman’s fertility, which declines with age [2, 37]. Borght et al. reported that female fertility already begins to decline around the age of 25–30 in most of the study population that experienced natural fertility [2]. Our stratified analysis showed that the association between uric acid and infertility was only found in women aged > 30 years. The results suggested that serum uric acid levels may be a useful indicator of association with infertility in women aged > 30 years. Future prospective studies are needed to further explore the causal association between serum uric acid and female infertility.

This study investigated the association between serum uric acid levels and female infertility based on nationally representative data from NHANES. In addition, the current study conducted a stratified analysis based on factors affecting female infertility such as BMI and age. However, several limitations of this study should be considered. Firstly, the specific causes of infertility cannot be identified due to the limitation of NHANES, and the association between serum uric acid and different causes of infertility needs to be further investigated. Secondly, the causal relationship between serum uric acid levels and female infertility could not be established due to the cross-sectional study design of this study. Thirdly, NHANES data collection through the form of questionnaire may have recall bias affecting the accuracy of the results.

## Conclusions

This study explored the relationship between serum uric acid levels and female fertility based on data from NHANES. The results found that women with high serum uric acid levels were associated with higher odds of infertility, and this association may vary by BMI and age. Serum uric acid levels may be a useful indicator of female infertility, but further confirmation of the association between serum uric acid and female infertility is needed.

## Data Availability

The datasets generated and/or analyzed during the current study are available in the NHANES database, https://wwwn.cdc.gov/nchs/nhanes/.

## Abbreviations

PCOS: polycystic ovary syndrome
NHANES: National Health and Nutrition Examination Survey
NCHS: National Center for Health Statistics
RHQ: Reproductive Health Questionnaire
DCHBS: 5-dichloro-2-hydroxybenzene sulfonate
BMI: body mass index
